# Arbovirus Detection in *Aedes aegypti* Mosquitoes in Manabí, Ecuador

**DOI:** 10.3390/pathogens14050446

**Published:** 2025-05-01

**Authors:** Alvaro Wilca-Cepeda, Andrea López-Rosero, Cesar A. Yumiseva, Mario J. Grijalva, Anita G. Villacís

**Affiliations:** 1 Centro de Investigación para la Salud en América Latina, Facultad de Ciencias Exactas y Naturales, Pontificia Universidad Católica del Ecuador, Quito 170530, Ecuador; awilca078@puce.edu.ec (A.W.-C.); aclopezrosero@hotmail.com (A.L.-R.); cayumiseva@puce.edu.ec (C.A.Y.); grijalva@ohio.edu (M.J.G.); 2 Infectious and Tropical Disease Institute, Department of Biomedical Sciences, Heritage College of Osteopathic Medicine, Ohio University, Athens, OH 45701, USA

**Keywords:** *Aedes aegypti*, arboviruses, Manabí, molecular techniques, Ecuador

## Abstract

Arboviruses transmitted by *Aedes aegypti* pose a significant challenge in Ecuador, as they are persistent, emerging, and re-emerging. During the SARS-CoV-2 pandemic, these diseases were temporarily overshadowed. This study aimed to detect and identify arbovirus species in mosquitoes collected from two communities in Manabí province—Caja Fuego (rural) and San Gregorio (marginal urban). A total of 468 mosquitoes were collected—385 from Caja Fuego and 83 from San Gregorio—and grouped into 72 pools. Samples were transported to CISeAL under proper biosafety protocols, homogenized, and analyzed using End-Point PCR, RT-PCR, and Sanger sequencing. The results revealed the presence of Flaviviruses and Alphaviruses. Of the 22 Flavivirus pools tested, 18 were positive, with PCR analysis specifically identifying dengue and Zika. Although no arbovirus was identified via RT-PCR, Sanger sequencing detected two Zika-positive samples. Notably, no official Zika cases were reported in 2023, suggesting a potential undetected risk of infection in human populations of Caja Fuego and San Gregorio. This study recommends the implementation of a surveillance campaign in collaboration with the Ecuadorian Ministry of Public Health to mitigate the risk.

## 1. Introduction

Arboviruses represent a significant group of viral families, including the Togaviridae and Flaviviridae families, characterized by their spherical morphology and RNA-based genomes [1]. These viruses possess the ability to be transmitted by arthropod vectors, primarily mosquitoes of the *Aedes* genus. Viruses such as Zika (ZIKV), dengue (DENV), and chikungunya (CHIKV) are responsible for high morbidity and mortality rates globally [2].

The life cycle of *Aedes aegypti* unfolds through four distinct stages, egg, larvae, pupa, and adult, classifying it as a holometabolous species. However, the epidemiology of arboviral diseases is complex due to multiple contributing factors, including (i) *Aedes aegypti* females being the sole vectors for arbovirus transmission through their blood-feeding behavior [3], (ii) socioeconomic factors, (iii) climate change [4], (iv) natural disasters, and (v) human mobility, all of which contribute to the proliferation of mosquito populations [5,6]. Consequently, *Aedes aegypti* has been able to expand into new geographical regions, facilitated by temperature variations that enable the vector to colonize previously uninhabited environments [7].

The epidemiological importance of *Aedes aegypti* extends beyond its capacity to harbor various strains of dengue, Zika, yellow fever, and chikungunya viruses. It is also notable for its widespread distribution across tropical and subtropical regions of the Americas, Africa, and Asia [8]. According to the Pan American Health Organization (PAHO), the incidence of diseases transmitted by *Aedes aegypti* increased in the Americas from 2013 to 2017. Moreover, there remains a significant lack of effective control strategies to mitigate the spread of diseases like hemorrhagic fever and dengue fever, which are transmitted by this mosquito [9]. In comparison to other diseases, global cases of dengue, Zika, and chikungunya have risen by 17%, with untreated cases contributing to an estimated 1 million deaths annually [10]. In the Region of the Americas, the total number of arboviral disease cases reported in 2022 up to epidemiological week 52 represents a relative decrease of 118.5% compared to the same period in 2021 [11,12]. Among vector-borne diseases (VBD), dengue remains the most fatal arboviral disease in the Americas, with outbreaks occurring every 3 to 5 years [12]. The number of reported dengue cases in the Americas surged in 2018, with Ecuador recording 8419 cases in 2019, of which 1718 (20.47%) presented warning signs. The case fatality rate stood at 0.044% [13].

In 2022, Ecuador documented 16,017 confirmed dengue cases, with 88.23% of cases showing no warning signs, 11.08% presenting warning signs, and 0.68% classified as severe. The circulating dengue serotypes were DENV-1 and DENV-2. Between 2014 and 2020, the province of Manabí reported a dengue rate of 84.61 cases per 100,000 inhabitants [13]. Zika, which entered the Americas in 2014, led to a rise in microcephaly cases, with Ecuador recording 4180 such cases by 2016. The prevalence of Zika surged by 85% following the 2016 earthquake [14]. The chikungunya virus, which was first reported in the Americas in 2013, resulted in over 1.3 million infections across 43 countries. Ecuador experienced a peak of 33,619 cases in 2015, with a dramatic decline in subsequent years, and no cases were reported in 2022. Despite *Aedes aegypti* being the vector for both Zika and chikungunya, no cases of either virus were recorded in Ecuador in 2022 [15].

Climatic changes have played a role in the expansion of *Aedes aegypti* populations, thereby enhancing the potential for widespread transmission of arboviral diseases [4]. Transmission has also been observed in regions previously uninhabited by these mosquitoes, such as the mountainous areas of Loja [16].

While research in Ecuador has primarily focused on aspects of mosquito ecology and resistance to insecticides used for vector control, there has been limited investigation into arbovirus transmission in areas affected by these diseases. Noteworthy studies include those addressing the high prevalence of Zika virus in *Aedes aegypti* populations in southwestern Ecuador [17], as well as research into seasonal and geographic variations in insecticide resistance in *Aedes aegypti* in southern Ecuador [18] and the resistance of *Aedes aegypti* to deltamethrin, malathion, and temephos in the region [19].

The present study aims to detect and identify various arboviral species present in *Aedes aegypti* mosquitoes collected from two communities—Caja Fuego and San Gregorio—located in Manabí province. The overarching objective of this study is to reduce infection risks in Manabí and increase awareness among local populations in these endemic regions. In many marginalized areas, a lack of financial resources, inadequate infrastructure, and limited access to technology and data contribute to the altered patterns of tropical diseases. Consequently, the surveillance and data collection efforts in such areas fail to accurately reflect the real-world situation in developing countries.

## 2. Material and Methods

### 2.1. Study Area

**Figure 1:**
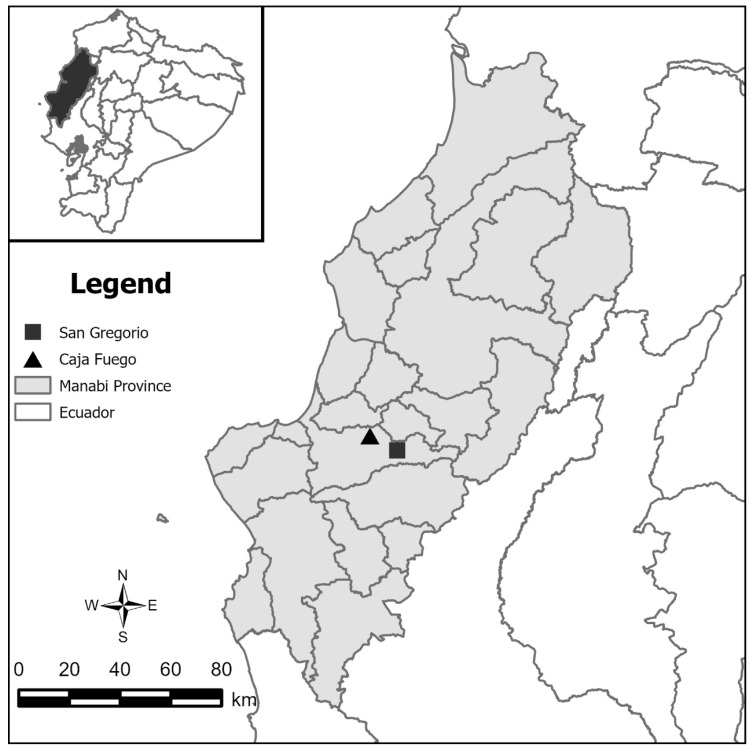
Map of Manabí, Ecuador. Black triangle represents Caja Fuego, rural community. Black square represents Andrés Vera, San Gregorio-marginal urban; both locations are found in Portoviejo County.

The samples used in this study were collected in Manabí province, in two communities, Caja Fuego (rural community and Andrés Vera (San Gregorio—marginal urban) ranging from 65 to 400 m above sea level (masl), located in a single county, Portoviejo (Figure 1).

Manabí province, located in Ecuador, has a tropical and subtropical climate and presents six distinct vegetation zones: deciduous forest, semi-deciduous forest, low mountain green forest, cloud forest, dry-montane shrub forest, and tropical Savannah [20]. Agriculture, particularly sugar cane, oranges, bananas, yucca, corn, rice, and palms like cade and coconuts, is the main economic activity in rural areas. Traditional houses are often built with bamboo cane (caña guadúa) or wood for walls and floors, with roofs being made of cade palm fronds [21,22]. In contrast, houses in marginal-urban areas typically have brick walls, cement floors, and zinc roofs [23].

### 2.2. Mosquito Collection

The *Aedes aegypti* mosquito collection was conducted in May 2023 during the rainy season. This was carried out with a collection permit (MAAE-DBI-CM-2021-0185) and a mobilization permit (MAAE-CMARG-2020-0178). The houses for collection were selected and mapped using a drone and georeferenced with GPS, assigning a unique code to each site.

The mosquitoes were collected using (i) electric aspirators, specifically Prokopack, due to its suction power, lightweight nature (0.5-kg), low rate of power consumption (4.3 amps), and water resistance. A 4:3-inch flexible rubber coupling (PlumbQuick model P1056-43, Fernco Inc., 300 S. Dayton St, Davison, MI, USA) [24] and (ii) manual aspirators collected specimens through a filter-equipped mouthpiece, ensuring that the mosquitoes remained in good condition.

A stereoscope was used in the field to identify *A. aegypti* specimens and determine their sex. The collection was conducted with support from the Centro de Investigación para la Salud en América Latina (CISeAL) and the Ecuadorian Ministry of Public Health (MSP).

Subsequently, the mosquitoes were grouped in sets of 10 and placed in tubes containing 1000 μL of TRI Reagent^®^ solution. It is worth noting that the tubes with mosquitoes were kept at optimal temperatures (4 °C) for further processing in the laboratories of the CISeAL.

### 2.3. RNA Extraction in Pool

To homogenize the mosquitoes, plastic pestles were used while following TRI Reagent^®^ guidelines to maximize RNA yield (TRIzol Reagent User Guide-Pub. no. MAN0001271). The RNA pellets were washed three times: First, 200 μL of chloroform was added per 1000 μL of Trizol, mixed, incubated for 15 min, and centrifuged at 12,000 RPM for 15 min at 4 °C. The supernatant was transferred to a new tube. Next, 800 μL of isopropanol (C_3_H_8_O) was added to 100 μL of supernatant, vortexed, incubated at −80 °C for 30 min (the original −70 °C temperature of the TRIzol Reagent guideline was changed to −80 °C because this was shown to improve pellet yield), and centrifuged at 12,000 RPM for 30 min at 4 °C, forming a new pellet. The pellet was then washed with 1000 μL of 75% ethanol, vortexed, and centrifuged at 7500 RPM for 5 min at 4 °C. Finally, the pellet was resuspended in 50 μL of nuclease-free water. Two aliquots were taken: 35 μL stored at −80 °C and 15 μL stored at −20 °C for future analysis [17].

### 2.4. cDNA Synthesis

The extracted RNA samples were converted to cDNA using the Promega-GoScript Reverse Transcription Kit. The process had two stages: First, a mixture of 2 μL nuclease-free water, 1 μL of random primers, and 2 μL of RNA sample was heated at 70 °C for 5 min; frozen at 4 °C; and centrifuged for 10 s. In the second stage, a retrotranscription mix was prepared with 6.5 μL of nuclease-free water, 4 μL of GoScript 5× reaction buffer, 2 μL of MgCl, 1 μL of PCR nucleotide mix, 0.5 μL of RNAase, and 1 μL of GoScript reverse transcriptase. The retrotranscription mix was combined with the linearized RNA (final volume 20 μL) and placed into a thermo-cycler with the following settings: 25 °C for 5 min, 42 °C for 1 h, and 70 °C for 15 min. The resulting cDNA was stored at −80 °C for future analysis [17].

After synthesizing and preserving the cDNA at −80 °C, three techniques were used for the identification of arbovirus: (i) End-Point PCR, (ii) Real-Time PCR, and (iii) Sanger Sequencing.

### 2.5. Positive and Negative Controls

Previously identified arbovirus samples stored in the CISeAL database were used as positive controls: 3M-05-D (dengue control), KEN C-19 (Zika control), and CHK 1752 (chikungunya control). Samples were re-amplified and aliquoted. In addition, 20 µL of PCR reaction mixture without template DNA was used as a negative control.

### 2.6. End-Point PCR

For Flavivirus detection, a reaction mix was prepared with 11.25 μL of nuclease-free water, 5 μL of buffer 5X green, 2 μL MgCl_2_ mM, 0.5 μL of nucleotide mix, 10 mM, 2.5 μL of primer forward (CFD2), 25 of primer μL reverse (MA) (Table 1), 0.25 μL GoTaq, and 1 μL of sample. The amplification was performed in a thermo-cycler with the following settings: 94 °C for 2 min, 35 cycles of 94 °C for 45 s, 55 °C for 1 min, and 72 °C for 1 min, followed by 72 °C for 7 min [25]. For dengue detection, the same protocol and thermo-cycler settings were used, but with different primers (D1-F), as well as for the reverse (D2-R) (Table 1) [26]. For Zika detection, the reaction mix was adjusted to 8.34 μL of nuclease-free water, 3.8 μL of primer forward (ZIKVENVF), and 4.11 μL of primer reverse (ZIKVENVR) (Table 1), and the thermo-cycler settings were modified as follows: 95 °C for 2 min, 35 cycles of 95 °C for 20 s, 55 °C for 20 s, and 68 °C for 30 s, followed by 68 °C for 7 min [27]. Finally, the PCR products for all viruses were visualized on a 1.5% agarose gel with ethidium bromide.

### 2.7. Real-Time PCR and Sanger Sequencing

For the detection of Zika, dengue, and chikungunya, a bioPerfectus multiplex RT-PCR kit was used, following the kit’s protocol with a Bio-Rad real-time detection machine [29]. After RT-PCR analysis, Sanger sequencing was performed to verify the results. Four independent reactions were conducted, each using the amplicon mixtures from the End-Point PCR analysis for dengue and Zika (Table 1), along with specific primers, four 2′-deoxynucleotides (dNTPs), and 4′-dideoxynucleotides (ddNTPs) in varying proportions. The resulting fragments were identified using a polyacrylamide gel [30].

### 2.8. Bioinformatic Analysis

After obtaining the results from Sanger sequencing, the synthesized sequences were analyzed using Geneious Prime software 2024.0. The process began with the general assembly of dengue and Zika sequences, which helped to identify the samples that successfully aligned and facilitated the cleaning of the sequences. Once the sequences were finalized, they were submitted to the National Center for Biotechnology Information’s (NCBI) BLAST version 2.15.0 nucleotide tool for identification.

## 3. Results and Discussion

The total mosquito collection in the two Manabí communities yielded 468 specimens, distributed into 72 pools. Of these, 385 mosquitoes were collected in Caja Fuego (62 males, 323 females) and 83 mosquitoes were collected in San Gregorio (40 males, 43 females). The collection was consistent with expectations for the rainy season. In End-Point PCR analysis, 11 positive samples for dengue and 7 for Zika were identified, but the RT-PCR did not detect any arboviruses. Among the 72 initial pools, only two Zika-positive pools (samples 21 and 53) were identified by Sanger sequencing. Their sequences were compared using BLAST, showing 91.81% and 94.20% similarity, respectively, to the MT483911.1 reference sequence, representing 2.78% of the samples—one from Caja Fuego and one from San Gregorio, both consisting of female *Aedes aegypti* mosquitoes. A comparison of the three techniques used for arbovirus identification in *A. aegypti* is summarized in Table 2.

The comparison between communities is limited because there are insufficient data due to the limitations of these marginalized areas. For this reason, this study aims to highlight the importance of the information, as the last more detailed study on these diseases was conducted in 2018 with geographic and demographic mapping in Central and South America; however, it did not detail the communities addressed in this study [31].

On the other hand, the literature mentions that the coinfection and cotransmission of these diseases in mosquitoes are limited by (i) infection enhancement, (ii) viral interference, (iii) the mosquito’s immune response, etc. [32]. Additionally, according to the study “Impact of simultaneous exposure to arboviruses on infection and transmission by *Aedes aegypti* mosquitoes”, the coinfection and cotransmission of arboviruses in mosquitoes can be replicated; however, this coinfection is only partially replicated in the laboratory, and it is not known how this coinfection may develop naturally [33].

Multiplex RT-PCR has been shown to be the most sensitive technique for detecting multiple viruses simultaneously [29], offering higher sensitivity and the ability to amplify several viruses at once compared to traditional serological methods [34]. However, the quality of the samples in this study was poor due to RNA degradation, which may have been caused by factors such as isolation with Trizol, transport time, laboratory processing time, and prolonged exposure to room temperature [35]. Furthermore, the kit used is a specific kit to be used with human blood serum. It should be noted that there is no specific kit on the market for the detection of arboviruses in mosquitoes, so when testing with the positive controls, amplification was obtained via RT-PCR. However, when working with the collected samples, adequate amplification was not obtained; the internal control (IC) of the kit identified possible problems in extraction. This may be due to the processing time of the samples, and the phenol possibly degraded the genetic material, as RNA is more unstable [29]. The objective of analyzing or identifying these arboviruses in *Aedes aegypti* is to provide early detection of the disease and thus provide data for epidemiological surveillance in collaboration with the authorities in the areas studied.

Due to having technical problems with the molecular RT-PCR technique but having had favorable results with End-Point PCR, we decided to perform sequencing of the possible positives obtained by molecular weight using agarose gels. The results from Sanger sequencing in this study, which identified two positive Zika samples, stand in contrast to the MSP report for Manabí province in 2023. While 99 dengue cases were reported, no cases of Zika were documented [15]. This discrepancy highlights a significant difference between the official report and the findings of this study, indicating that individuals in the mosquito collection zones may be at risk of Zika virus infection.

Based on previous observations, it appears that arthropod-borne virus disease monitoring in Ecuador is insufficient. Dengue remains the most prevalent, with the highest number of reported cases, overshadowing Zika and chikungunya. From an epidemiological perspective, dengue is the primary focus. However, the Pan American Health Organization (PAHO) reported around 8758 Zika virus cases across the Americas by June 2023 [12]. In contrast, chikungunya, with fewer cases reported by the Ministry of Public Health (MSP) and better control through a newly developed vaccine, circulates less widely, leading to fewer detected cases compared to dengue [36].

Climate change and climatic phenomena have caused the distribution of arboviruses to spread to areas where these diseases were not previously found [31]. As mentioned in the introduction, due to these diseases, *Aedes aegypti* has adapted in Loja, Ecuador, thanks to the increase in temperature.

To gain more insights into these arboviruses and enhance the studies using techniques such as End-Point PCR, RT-PCR multiplex, and Sanger sequencing, the following are recommended: (i) sample purification, (ii) increasing sample size and using CAP questionnaires, and (iii) conducting serology studies. In addition, to minimize RNA sample degradation, it is advisable to reduce the exposure time to phenols during sample handling [37]. These measures are essential for obtaining a more comprehensive understanding of the diseases and their key factors. In conclusion, the goal is to improve the understanding and efficiency of identifying dengue, Zika, and chikungunya in targeted populations without direct human intervention. This approach aims to reduce the risk of infection, raise awareness in communities, and enhance surveillance efforts in collaboration with the Ministry of Public Health (MSP).

## Figures and Tables

**Table 1:** The primers used in the three methods to detect different arboviruses.

	Primer Pair (Code)	Amplicon	Source
Flavivirus	MA: 5′-CATGATGGGRAARAGRGARRAG-3′ CFD2: 5′-GTGTCCCAGCCGGCGGTGTCATCAGC-3′	260 bp	[25]
Alphavirus	M2W: 5′-(CT)AGAGC(AGT)TTTTCGCA(CT)(GC)T(AG)GC(ACT)(AT) cM3W: 3′-ACAT(AG)AAN(GT)GNGTNGT(AG)TC(AG)AANCC(AGT)A(CT)CC	434 bp	[26]
dengue	D1-F: 5′-TCAATATGCTGAAACGCGCGAGAAACCG-3′ D2-R: 5′-TTGCACCAACAGTCAATGTCTTCAGGTTC-3′	511 bp	[27]
Zika	ZIKVENVF: 5′-GCTGGDGCRGACACHGGRACT-3′ ZIKVENVR: 5′-RTCYACYGCCATYTGGRCTG-3′	364 bp	[28]

**Table 2:** The results of the different arboviruses found with the three applied techniques.

	End-Point PCR	RT-PCR Kit (bioPerfectus)	Sanger Sequencing
Flavivirus	>75%	N/A	N/A
dengue	11	0	0
Zika	7	0	2

## Data Availability

The data presented in this study are available on request from the corresponding author.
